# Supplementary figures and images for: Corneal biomechanical predictors of intraocular pressure elevation after intravitreal anti-VEGF injection
Source: PLoS One. 2025 Aug 22;20(8):e0330574. doi: 10.1371/journal.pone.0330574 (PMC12373209; doi:10.1371/journal.pone.0330574)

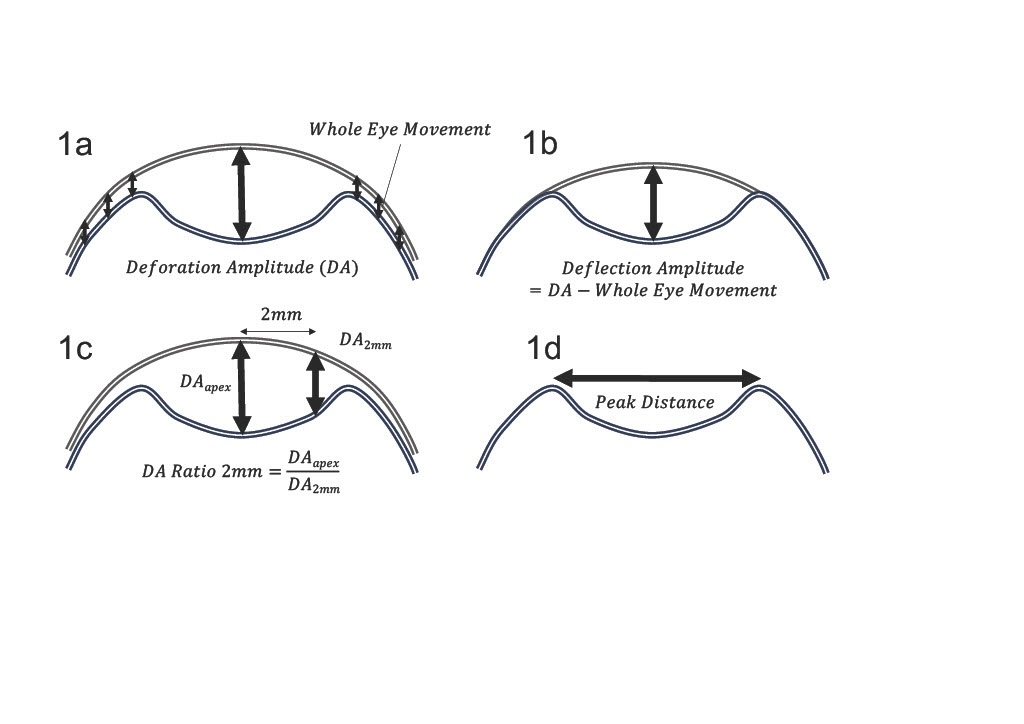

Supplement: S2 Fig — (TIF) [file pone.0330574.s002.tif]
