# Supplementary material for: Corneal biomechanical predictors of intraocular pressure elevation after intravitreal anti-VEGF injection
Source: PLoS One. 2025 Aug 22;20(8):e0330574. doi: 10.1371/journal.pone.0330574 (PMC12373209; doi:10.1371/journal.pone.0330574)
Supplement: Attachment — Submitted filename: renamed_b5493.docx [file pone.0330574.s003.docx]

### ****General Assessment****

This retrospective study investigates corneal biomechanical parameters as predictors of acute IOP elevation post anti-VEGF injection, addressing a clinically relevant complication. The study design is appropriate, and the use of Corvis ST for biomechanical assessment is well justified. However, several methodological and conceptual limitations need addressing to strengthen the manuscript.

**Major Comments**

1. The sample size (40 eyes) is modest, and no power calculation is provided. Given the retrospective nature, please discuss whether the sample size was sufficient to detect clinically meaningful differences in IOP elevation (e.g., ≥10 mmHg spikes).Consider stratifying results by diagnosis (nAMD vs. RVO) to assess disease-specific effects, as these conditions may differ in baseline ocular rigidity.
2. Clarify why other Corvis ST parameters (e.g., SSI) were not significant predictors despite their known association with corneal rigidity.
3. The proposed cutoff for DA Ratio MAX (≤4.936) has high sensitivity but low specificity (42.9%). Discuss how this trade-off impacts clinical utility. Would combining it with other parameters (e.g., baseline IOP) improve predictive value?
4. Report how missing data (if any) were handled. For example, were eyes with prior glaucoma or corneal pathology excluded? Control for potential confounders such as axial length (not mentioned), which influences ocular biomechanics.

### ****Minor Comments****

1. Standardize terms: "IOP elevation" vs. "IOP spike" should be clearly defined (e.g., ≥10 mmHg threshold).
2. Report confidence intervals for β coefficients in multivariate analysis (e.g., β = −5.40 for DA Ratio MAX).
3. Corneal biomechanical parameters, as currently recognized indicators, need to be compared with the findings of PMID: 38241831 on post-injection intraocular pressure (IOP) and corneal biomechanics to strengthen the persuasiveness of this study.
4. The dynamic corneal response parameters measured by Corvis ST, as emerging metrics, should be compared with the detection results of other biomechanical assessment devices, such as the Ocular Response Analyzer, to enhance their persuasiveness in clinical applications.
